# A Program to Improve Digital Access and Literacy Among Community Stakeholders: Cohort Study

**DOI:** 10.2196/30605

**Published:** 2021-11-10

**Authors:** Brittany F Drazich, Yeukai Nyikadzino, Kelly T Gleason

**Affiliations:** 1 School of Nursing Johns Hopkins University Baltimore, MD United States

**Keywords:** technology, disparities, digital access, digital literacy, community, stakeholders, digital health, digital divide, patient-centered outcomes

## Abstract

**Background:**

For many research teams, the role of community stakeholders is critical. However, community stakeholders, especially those in low-income settings, are at risk of being excluded from research and community engagement initiatives during and after the COVID-19 pandemic because of the rapid transition to digital operations.

**Objective:**

We aimed to describe the implementation and feasibility of a program called Addressing the Digital Divide to Improve Patient-Centered Outcomes Research, which was designed to address barriers to technology use, and to examine changes in participants’ perceived comfort with digital technology before and after the program.

**Methods:**

To promote full engagement, we worked with 20 existing community leaders to cocreate a training course on using digital technology. We assessed the frequency of technology use and comfort with technology through an adapted 8-item version of the Functional Assessment of Comfort Employing Technology Scale and used the Wilcoxon signed-rank test for survey analysis. We also conducted a focus group session with 10 participants and then performed reflective journaling and content analysis to determine emergent themes.

**Results:**

We found that the program was feasible to implement and worthwhile for participants (15/16, 94%). After the program, the participants perceived an increase in the frequency of technology use (z=2.76, *P*=.006). The participants reported that the program was successful because of the technology training program, but recommended that the program have a slower pace and include a helpline number that they could call with questions.

**Conclusions:**

Future programs should consider that populations with low literacy view technology training as a core element to decreasing technology disparity. This study demonstrates that through low-cost input, community members can be provided the resources and training needed to virtually participate in research studies or community engagement initiatives.

## Introduction

In an effort to mitigate the spread of disease and prevent unnecessary death caused by the COVID-19 pandemic, public health leaders and policymakers globally have recommended or instituted physical distancing measures [1-4]. Subsequently, people have replaced in-person interactions with virtual interactions [5-9]. For example, in the United States, the use of telehealth, virtual learning, telework, and personal video chat communication has increased [10-13]. This use of technology has allowed people to remain active and engaged with others during the pandemic.

Research teams, many of which include community members, have also adapted operations to virtual platforms such as videoconferencing wherever possible. For many research teams, the role of community stakeholders is critical. Community stakeholders can assess the importance and cultural relevance of a research question. Involvement of community stakeholders in research can improve recruitment and sustainability of outcomes [14]. From an ethical standpoint, many assert that the community has a right to be aware of and involved in research that could affect them [15]. Community stakeholders involved in research are often leaders in their communities who facilitate connections and ensure awareness of community resources [15-18].

Although technology has the potential to assist community stakeholders in remaining engaged during the COVID-19 pandemic, there are technology-based disparities [19-21]. Many community stakeholders, especially those in low-income settings, are at risk of being excluded from research and community engagement initiatives during and after the COVID-19 pandemic because of the rapid transition to digital operations. In Baltimore, Maryland where this study was conducted, an estimated 40% of households do not have wireless internet service and one-third of households do not have a desktop or laptop computer [22-24]. Black Americans, who make up 67% of the population of Baltimore [25], experience more barriers to technology use than the rest of the city’s population because of systemic and structural racism that has led to socioeconomic disparities [26,27]. Black older adults experience additional barriers to technology use such as lack of guidance, lack of confidence, limited resources, and the perception that technology is complex [28,29].

These barriers to technology use severely restrict Baltimore’s community members from getting involved in research teams and supporting their communities during the COVID-19 pandemic. It is essential to address technology-based disparities, or the “digital divide,” to ensure full community engagement for patient-centered research design and generalizable research samples both during and after this pandemic. This study aimed to describe a program called Addressing the Digital Divide to Improve Patient-Centered Outcomes Research or ADD2PCOR, which was created to address the barriers to technology use. Through this program, we aimed to provide a time-efficient, cost-effective, and feasible training course to bridge the digital divide among Baltimore’s community stakeholders and impart technological knowledge and tools to those who would otherwise be unable or unlikely to participate digitally. In this paper, we describe the implementation and feasibility of ADD2PCOR and examine participants’ comfort with digital technology before and after the program.

## Methods

### Project Description

#### ADD2PCOR Goals

We co-designed, implemented, and refined ADD2PCOR to provide community stakeholders and vulnerable patients with basic technological knowledge and equipment that would help them to participate virtually. We intended for this program to be both cost- and time-efficient so that it could be broadly implemented by research teams, both within our institution and elsewhere, who wish to engage people with low technology literacy in research activities.

#### Participants and Recruitment

We recruited participants who were 18 years or older, participated as a community member on a research advisory council or similar committee or confirmed intent to join a research advisory council or similar committee in the coming year, and verbalized that they did not have regular access to internet at their home or a digital tool beyond their phone to access the internet. We performed snowball sampling through our existing partnerships with patients and the community and recruited 20 community stakeholders.

#### Training Course Development

To promote full engagement, we worked with existing community leaders to cocreate a training course on using digital technology. Brevity and feasibility were emphasized throughout training course creation to ensure that other organizations may be able to easily implement the course without the need to devote significant resources. We obtained input from community and patient groups such as the Community Research Advisory Council, the Patient and Family Advisory Council, and Patients Aligned with Research Teams and ER Nurses to Improve Diagnosis to understand the difficulties that patients and community members have faced in engaging in virtual meetings and using other basic technologies. Training course development in this project followed the classic 4 elements of the Tyler Model of planning, designing, implementing, and evaluating with community stakeholders engaged in the conception of each aspect [30]. Codevelopment is the cornerstone of the design portion of this curriculum development. We created written material to loosely guide training course progression for both the trainer and community stakeholders. All learning topics in the training course were determined based on community stakeholder input, which were then amended based on individual participant needs during program implementation.

#### Project Implementation

Each community stakeholder recruited for this study received an Amazon Fire tablet, which was purchased at a price as low as $40. If they did not already have broadband internet access in their home, they were given a 1-year Comcast Internet Essentials plan, which was available for $9.95 a month inclusive of setup and rental costs [31]. The maximum cost of providing both internet access and a tablet was $200 a year, which is comparable to that spent on meals and parking for participants in many research or engagement programs.

Based on suggestions from community stakeholders, the first section of the training course focused on ensuring that participants were comfortable with the basic functions of the tablet, including switching on the tablet, charging the tablet, and connecting to the internet. Once we confirmed that participants were able to access the internet, we introduced free basic services such as Gmail. We ensured that participants could open their email accounts as well as documents and calendar invitations sent through email. The second section of the training course focused on ensuring that participants were comfortable using videoconferencing and at least one application (or “app”) of their choosing. For example, many participants requested assistance in learning how to access their patient portal for health information or use social media to view pictures of their grandchildren. After the majority of participants were confident with both connecting to the internet and videoconferencing, we conducted 1-hour video group meetings to review common areas of difficulty with the use of the tablet. Participants who had not yet mastered videoconferencing joined the video meetings via phone-based audio. These group meetings were opportunities for the community stakeholder participants to learn from each other or solidify learned skills.

**Figure 1:**
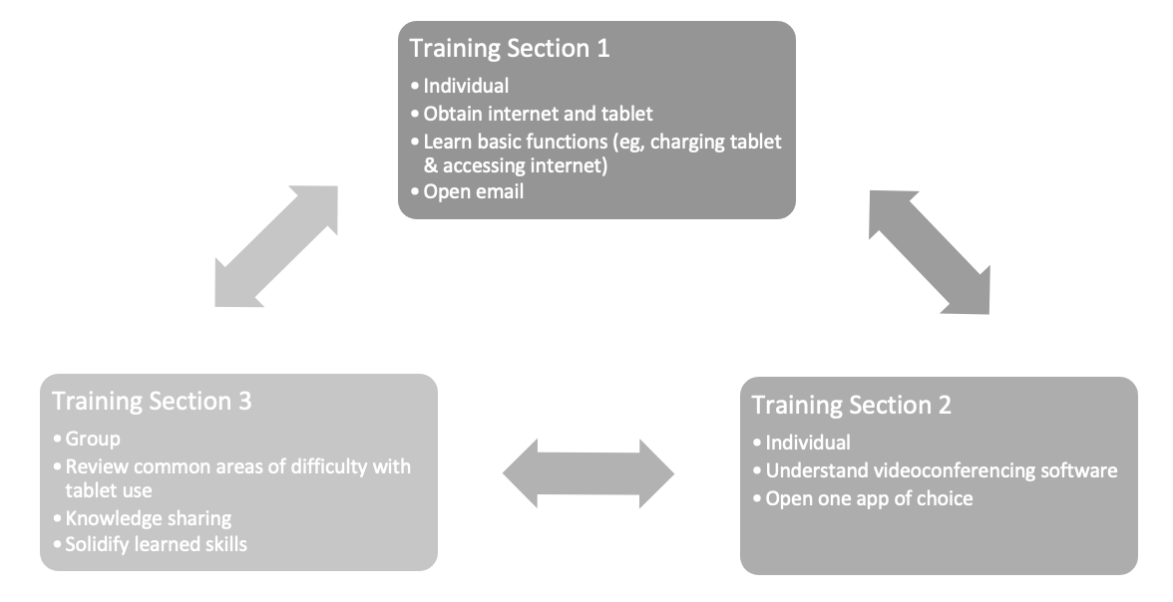
Example of a community stakeholder training course. The arrows indicate the iterative nature of the training course.

We compensated participants $30 for each hour of training that they attended. Although the training course was originally planned as in-person instruction, we delivered all training sessions virtually because of pandemic-related safety concerns. Similarly, although the training course was originally planned as group-based learning, the majority of the training course was delivered through one-on-one instruction over phone or video chat. We determined one-on-one instruction to be more efficient following the switch to virtual instruction because it allowed the delivery of individualized education and limited extraneous background noise during video meetings. Although we originally planned for the training course to be 3 hours long for all participants, we changed this to a more flexible format during the program based on participant needs and feedback. However, on average, participants completed 3 hours of training course content. Although the training course components and pace were individualized based on the technology literacy and preferences of each community stakeholder, a step-by-step example is described in Figure 1.

### Data Collection and Analysis

We used Qualtrics, an online survey platform, to understand the frequency of use of various digital technologies by community stakeholders before and after ADD2PCOR. We considered frequency of technology use to be an indicator of comfort with technology. We assessed frequency of technology use through an adapted 8-item version of the Functional Assessment of Comfort Employing Technology Scale (FACETS). FACETS encompasses 5 functional domains: social, e-commerce, technical, health care, and home [32]. An example survey question is “I use Google or another search engine to find answers to questions.” Scores for participants’ responses to each question ranged from 0 to 6, with higher scores indicating a greater frequency of technology use [32]. The survey responses were combined for an overall score ranging from 0 to 48. The adapted FACETS survey is shown in Multimedia Appendix 1. We used the Wilcoxon signed-rank test to compare responses on the frequency of technology use before and after ADD2PCOR, considering a *P* value <.05 as significant.

After all community stakeholders participated in the training course, we conducted a focus group session with the ADD2PCOR participants who expressed an interest in joining it. The purpose of the focus group was to receive feedback from the participants on their experience with ADD2PCOR. The researcher who moderated the focus group discussion was an experienced qualitative interviewer. In an effort to prevent bias, the moderator and notetaker present had no prior interactions with the ADD2PCOR participants. Questions in the semistructured interview guide were related to previous comfort with and use of digital technology, motivation to join ADD2PCOR, positive and negative feedback on ADD2PCOR, opinion on the training aspects of the program, and comfort with and use of digital technology after ADD2PCOR. The focus group session was conducted as a virtual video meeting of 10 participants that lasted approximately 1 hour and 15 minutes.

Although the focus group meeting was not recorded, thorough notes of direct quotations were taken from participants throughout the session, followed by reflective journaling and content analysis conducted in a manner similar to that by Halcomb and Davidson [33]. We selected content analysis for the organization of focus group data because of our study’s focus on obtaining objective feedback for program improvement. The focus group moderator (BFD) utilized the coding software f4analyze (audiotransckription) to organize direct quotations, and the project lead (KTG) reviewed the content analysis and field notes and validated the selected themes [34].

## Results

### Characteristics of the Sample

In total, 16 of the 20 community stakeholder participants (85%) completed the demographical survey (Table 1) and the FACETS survey (Table 2). The majority of the participants were female and aged 65-74 years. All study participants identified as Black individuals. The majority of participants were extremely satisfied with the program (11/16, 69%) and strongly agreed that the experience was worthwhile (15/16, 94%).

**Table 1:** Sample characteristics.

Characteristic			Participants (N=17), n (%)
**Age (years)**			
	45-54		1 (6)
	55-64		3 (18)
	65-74		10 (59)
	75-84		2 (12)
	>85		1 (6)
**Race**			
	Black	17 (100)	
**Gender**			
	Women	14 (82)	
	Men	3 (18)	
**Education level**			
	Less than high school	2 (13)	
	High school graduate	4 (25)	
	Some college	6 (38)	
	4-year degree or more	5 (29)	
**Satisfaction level^a^**			
	Extremely satisfied	11 (69)	
	Somewhat satisfied	5 (31)	
**How likely recommend to a friend^a^**			
	Extremely likely	14 (88)	
	Somewhat likely	2 (12)	
**Found experience worthwhile^a^**			
	Strongly agree	15 (94)	
	Somewhat agree	1 (7)	

^a^n=16.

**Table 2:** Changes in technology use frequency (N=17).

FACETS^a^ domains		Score, mean (SD)		z value	*P* value
**Texting frequency**				−2.18	.03
	Pre	4.15 (1.90)			
	Post	4.88 (1.68)			
**Social media use frequency**				−1.23	.22
	Pre	2.25 (1.62)			
	Post	3.38 (1.89)			
**Wi-Fi use frequency**				−2.75	.01
	Pre	3.25 (1.62)			
	Post	5.13 (1.41)			
**Videoconferencing frequency**				−2.73	.01
	Pre	2.45 (1.73)			
	Post	4.81 (1.64)			
**Searching on Google frequency**				−2.17	.03
	Pre	4.35 (1.63)			
	Post	5 (1.80)			
**Opening files frequency**				−1.04	.30
	Pre	2.45 (1.76)			
	Post	3.62 (2.30)			
**Opening shared files frequency**				−2.04	.04
	Pre	1.90 (1.86)			
	Post	3.5 (2.34)			
**Opening calendar invites frequency**				−3.20	.001
	Pre	1.45 (1.39)			
	Post	4.19 (2.04)			
**Combined frequency score**			−2.76		.006
	Pre	24.40 (11.05)			
	Post	38.31 (10.64)			

^a^FACETS: Functional Assessment of Comfort Employing Technology Scale.

### Survey Results for Technology Use

Cronbach α was .88 for the adapted FACETS survey. After comparing the FACETS measure before and after ADD2PCOR, we found a significant increase in the score for the following 6 subdomains of frequency of technology use: texting, Wi-Fi, videoconferencing, opening files sent by others, searching on Google, and opening calendar invites. The subdomains with the greatest increase in score on a scale of 0-10 were “videoconferencing frequency,” with an average increase of 2.36, and “opening calendar invites frequency,” with an average increase of 2.74. A comparison of the combined total subdomains scores before and after the program showed an increase in score by 13.91 (scale 0-48) for the frequency of technology use.

### Focus Group Results

During focus group analysis, we identified 3 major themes: motivation and benefits of the program, training as the core to success, and areas for program improvement. The findings are summarized below.

#### Motivation and Benefits of the Program

The participants overwhelmingly joined ADD2PCOR because they wanted “to learn.” Some participants reported that they generally “like to learn new things,” whereas others perceived a specific gap in knowledge regarding technology. The participants discussed that having skills in technology gives them comfort because technology can provide entertainment or enable them to contact others when in need. For example, one participant stated,

[Without this program],  I would be where I was before I started (laughs). You have broadened my resource base, gave me more tools to work with, to navigate the pandemic with.

Some of the participants reported that they had limited knowledge on affordable internet and device options before the program and that they intended to continue with the internet plan through the year after study conclusion. The participants were especially appreciative of being able to video chat with loved ones who they would otherwise not be able to see face-to-face because of the pandemic or geographical distance. Finally, the participants said that the program allows them to better serve their community during the pandemic. For example, one participant stated that,

[The program] allows us to dig into more resources for the community. People are isolated during the pandemic…We are community people. We want to continue doing what we do – getting a hold of resources and sharing them. The ultimate goal is helping others.

#### Training as the Core to Success

Most participants reported that the most valuable aspect of the program was the training or classes. The participants described how ADD2PCOR would be “useless” without the training classes, as their tablet “would just be lying there.” One participant narrated their experience of acquiring a new iPad, only to later give it away because he did not understand how to use it. Another participant described how her family members use technology on her behalf instead of offering to teach her how to use it. The participants expressed appreciation for having an instructor for the tablet who is patient with basic questions or slow progress. For example, one participant stated, “y’all take your time and take me step by step to learn the steps without getting frustrated.” The participants stated that receiving the tablet and receiving training to use it “go hand in glove”; each is imperative for the success of the other.

#### Areas for Program Improvement

The survey results indicated that the participants were extremely satisfied with ADD2PCOR; however, they did make some suggestions for similar future programs during the focus group session. Some participants suggested a slower pace of training “so we can learn every icon that pops up on our program and how to use it, what your tablet can do.” Another participant suggested that periodic recaps on past learned skills be offered for adults aged 80 years and older. Two older participants suggested that older adults, especially those with poor eyesight, be taught to use audio virtual assistants such as Alexa or audiobooks. The participants also expressed the importance of a helpline to call with questions outside of formal training meetings. Similarly, one participant recommended that veteran participants be partnered with newer participants to help troubleshoot problems with basic tablet functions.

## Discussion

Addressing disparities in technology access and use among community stakeholders is essential to creating and implementing culturally competent research studies and interventions. ADD2PCOR addressed this need by providing community stakeholders with a tablet and internet as needed and by implementing a self-paced technology training course. We found that ADD2PCOR was feasible to implement and worthwhile (15/16, 94%) for participants. After the program, the participants perceived an increase in the frequency of technology use in 6 subdomains: texting, Wi-Fi, videoconferencing, opening files sent by others, searching using Google, and opening calendar invites. The participants largely joined the program “to learn,” and believed that the greatest benefit of the program was that the skills they learned will help them obtain and provide help to others. The participants reported that the program was successful because of the technology training program, but recommended that the pace of the program be reduced and that a helpline number that they could call with questions be provided.

Considering the small sample size of this study, it is promising that participants’ frequency of technology use significantly improved in the majority of tested subdomains. The subdomains that showed a significant increase in score were those of core skills taught during the training or practiced by participants throughout the study, except for text messaging. Improving comfort with the use of technology in other areas may have translated to improvement in comfort with text messaging. These skills that showed a significant increase in score are critical skills needed for the community stakeholders to continue their involvement in research projects or community engagement in the virtual environment. While social media use increased among participants, there was no significant increase in the score for this subdomain. During the intervention, the training focused more on basic tablet functions than on the use of social media, per participant request. During the focus group session, the suggestion that the pace of training be reduced was provided mainly by older participants. Future studies on technology training might benefit from creating homogenous subgroups for training divided by baseline technology knowledge. Except for a few reports [35,36], most studies on technology-based interventions have focused on either training or improving access [37-39]. Our study results suggest that both are core elements for improving comfort with and use of technology and should be addressed in tandem.

Most of the participants in this study were aged 65 years or older and identified as Black individuals, which reflects the demographics of populations experiencing disparity in technology use and access in the United States. Many older Black Americans experience intersectional barriers to technology use such as inexperience arising from age-related preferences or disability [20,40] or financial constraints related to structural inequities [41]. During the COVID-19 pandemic, such technology disparities might contribute to poor vaccine uptake or limited health care options for these populations [42]. For example, COVID-19 vaccine scheduling and telehealth appointments are both health care activities that require technology-based literacy. Technology should be used as a tool to decrease and not perpetuate disparities [38,39,43-45].

During the pandemic, studies are being adapted to virtual platforms and this will likely continue after the pandemic. It is essential that all people have the resources and knowledge needed to engage in such virtual studies. Broadband internet and device ownership is less common in certain populations such as older adults or low-income populations [46,47]. Unfortunately, many virtual studies and interventions, even those aimed at decreasing disparities, require such resources for participation [37,45,48]. This study demonstrates that research teams can provide participants with a tablet, internet, and training through low-cost input. The intention of some of the participants to continue with the internet plan through the year after study conclusion indicates the sustainability of the program.

The major limitations of this study were the sample size, sampling procedure, and groupthink. The small sample size was appropriate for this feasibility study but might have had limited power in detecting smaller differences. In additional, we used a snowball sampling technique that might have included participants that are not representative of other community stakeholders in Baltimore. Finally, even though the focus group session was moderated by a researcher who had no prior interaction with the participants, participant feedback was overwhelmingly positive. The participants might have felt pressured to respond in a manner similar to their peers (groupthink) [49]. Regardless of these limitations, this study has many strengths. With the inclusion of community leaders and existing collaborations, we were able to recruit people who might have otherwise been difficult to reach through research. Furthermore, this study was able to implement participation feedback at all stages, thus providing individualized training as needed and making adjustments when components were found to be unsuccessful.

The findings of this study highlight areas in need of future research and policy change. This study demonstrates a feasible intervention that improves comfort with and use of technology for community stakeholders. Many previous researchers have excluded otherwise eligible participants from studies because of a lack of internet access or devices needed to participate in the study. The results of this study indicate that through low-cost input, community members can be provided such resources and be included in technology-based studies. Future studies could implement a program similar to ADD2PCOR in a larger sample and integrate the constructive feedback presented in the focus group. This study suggests that providing the necessary technological equipment alone might not be sufficient for improving technology use in all populations; researchers must also consider a participant’s sociotechnical environment [50,51]. For example, this study’s sample of older Black Americans with low technology literacy viewed technology training and a technology helpline as important supports for technology use. Similarly, if researchers or organizations intend to provide group learning for community members or study participants, they should consider dividing groups by technology literacy so that the training pace complements each individual. Recently, the Federal Communications Commission began the Emergency Broadband Benefit program, which provides affordable broadband services to low-income Americans [52]. Researchers who work with community stakeholders could also explore currently available free online classes that are specifically designed to improve technology literacy. Including community members as leaders or participants in studies is critical, and the lack of internet, technological equipment, or technology literacy are modifiable factors that can be addressed by research teams.

## Appendix

Multimedia Appendix 1 Adapted Functional Assessment of Comfort Employing Technology Scale survey.

## Abbreviations

ADD2PCOR: Addressing the Digital Divide to Improve Patient-Centered Outcomes Research
FACETS: Functional Assessment of Comfort Employing Technology Scale
