# Supplementary material for: A Program to Improve Digital Access and Literacy Among Community Stakeholders: Cohort Study
Source: JMIR Form Res. 2021 Nov 10;5(11):e30605. doi: 10.2196/30605 (PMC8663502; doi:10.2196/30605)
Supplement: Multimedia Appendix 1 [file formative_v5i11e30605_app1.docx]

*Appendix A.* Adapted FACETS Survey

Question 1. I send text messages using a smart phone...

o Never

o A few times a year

o A few times a month

o Once a week

o A few times a week

o Daily

Question 2. I post on social media (e.g., facebook, twitter)...

o Never

o A few times a year

o A few times a month

o Once a week

o A few times a week

o Daily

Question 3. I connect to Wi-Fi when it is available...

o Never

o Tried, but it didn't work

o Got help but didn't work

o Only with help

o Myself, with difficulty

o Myself easily

Question 4. I join virtual conference calls using my tablet’s video function...

o Never

o Tried, but it didn't work

o Got help but didn't work

o Only with help

o Myself, with difficulty

o Myself easily

Question 5. I use Google or another search engine to find answers to questions...

o Never

o A few times a year

o A few times a month

o Once a week

o A few times a week

o Daily

Question 6. I find, open, and close files in my computer...

o Never

o A few times a year

o A few times a month

o Once a week

o A few times a week

o Daily

Question 7. I open files shared by the research team/council/committee I work with...

o Never

o Tried, but it didn't work

o Got help but didn't work

o Only with help

o Myself, with difficulty

o Myself easily

Question 8. I open and accept calendar invites on my tablet...

o Never

o Tried, but it didn't work

o Got help but didn't work

o Only with help

o Myself, with difficulty

o Myself easily
